# A large national cohort study exploring the association between small for gestational age birthweight and educational achievement in secondary school

**DOI:** 10.1007/s00431-026-07372-w

**Published:** 2026-09-02

**Authors:** Alexia K. Searchfield, Barry Milne, John M. D. Thompson

**Affiliations:** 1 Department of Paediatrics, Child and Youth Health, University of Auckland, M&HS Building 507 - Bldg. 507, 28 Park Ave, Grafton, Auckland 1023 New Zealand; 2 Centre of Methods and Policy Application in the Social Sciences, University of Auckland, Auckland, New Zealand; 3 Department of Obstetrics, Gynaecology and Reproductive Sciences, University of Auckland, M&HS Building 507 - Bldg. 507, 28 Park Ave, Grafton, Auckland 1023 New Zealand

**Keywords:** Small for gestational age, Fetal growth restriction, Education, School achievement

## Abstract

Small for gestational age (SGA) birthweight is an important predictor of childhood intelligence, yet longitudinal evidence on its association with school achievement is scarce. We aimed to explore the association between SGA birthweight and educational achievement in secondary school in a national linked data study of New Zealand adolescents. This population study included children born in New Zealand with gestational age, birthweight and sex information available between 1998 and 2005. This cohort was followed until July 31, 2024. Exclusion criteria were children born overseas, multiple births, and children who moved overseas prior to secondary school examinations. SGA birthweight was defined using the New Zealand Sex-Specific Population Birthweight Standard. Educational achievement was defined as the highest qualification attained in secondary school. The analysis included 372,519 children (*n* = 25,341 [6.8%] small-for-gestational-age [SGA] and *n* = 347,178 appropriate-for-gestational-age [AGA]). Compared with infants born AGA, in multivariable analysis, children born SGA were at an elevated risk of achieving no formal school qualification (aRR 1.36, 95% CI: 1.25–1.47) compared to finishing secondary school with the maximum qualification available. This risk was greater for those with severe-SGA birthweight (aRR 1.48, 95% CI: 1.29–1.70). There was a statistically significant interaction with very preterm and preterm birth, indicating that SGA birthweight has a differential association with achievement across gestational ages. Crude estimates were larger, suggesting large contributions from confounders.

*Conclusion*: Children born SGA had a slightly lower risk of achieving higher secondary school qualifications than their AGA peers. This risk is higher for those not achieving any formal qualification and increases with the severity of SGA. The interaction with preterm birth suggests that the relationship between SGA birthweight and education may differ for very preterm and preterm infants. Adjustment for confounders decreased the associations between SGA birthweight and educational achievement in secondary school, indicating the importance of familial and societal factors.

**Table:** 

**What is Known:** *• Few studies have looked at the association between SGA birthweight and achievement in school.* *• The relationship between SGA birthweight and achievement remains unclear, as studies seldom adjust for confounding variables.*	
**What is New:** *• This study used administrative data to explore the association between SGA birthweight and educational achievement in secondary school.* *• There appeared to be a small but statistically significant association between SGA birthweight and education in secondary school. The effect size is small and confounded by social determinants, but the number of children affected is not inconsequential at the population level, with SGA having a prevalence of 10%.*

**Supplementary Information:**

The online version contains supplementary material available at https://doi.org/10.1007/s00431-026-07372-w.

## Introduction

Small for gestational age (SGA) is considered a proxy for fetal growth restriction (FGR) and is attributed to infants born in the lowest 10th percentile of birthweight on a national standard adjusted for gestational age and infant sex [1]. SGA is a heterogeneous group of infants, with only a subset having experienced FGR. The remaining infants were considered constitutionally small (genetically predisposed to small stature). SGA birthweight, along with preterm birth and low birthweight, accounts for most of perinatal and postnatal mortality and morbidity; however, despite recent estimates suggesting that almost 20 million children are born FGR annually, SGA/FGR remains a largely under-researched area [2]. FGR can be challenging to diagnose, and the required measures (e.g. Doppler studies) for this were not available through the New Zealand national data register. Therefore, SGA birthweight was used as a measure of growth compromise during fetal development. However, this is an imperfect measure and will not completely capture the FGR population.

Historically, research on SGA birthweight has primarily explored perinatal and neonatal mortality; however, with increased survival of SGA neonates, due to advancements in medical intervention and growth monitoring, there has been a shift towards understanding childhood morbidities associated with SGA birthweight. This includes studies suggesting a potential link between SGA birthweight and cognitive functioning, demonstrated through lower intelligence quotients on a range of developmental tests in childhood, and more sparingly with impaired school performance [3–6].

Despite increasing research into intelligence and educational outcomes for SGA children, results remain contradictory. It is suspected that this is due to a combination of insufficient sample power [7], inconsistent control of confounders [7, 8], differences in the definition of intelligence and educational achievement [6, 9], differing birthweight thresholds for identifying small for gestational age [10–12], and the presence of comorbidities such as preterm birth [10, 13].

This research aimed to address some of these weaknesses and gain a deeper understanding of the relationship between SGA birthweight and educational achievement. The primary objective of this analysis is to determine if there is a significant relationship between SGA birthweight and achievement on standardised examinations in New Zealand secondary schools. Secondary objectives include analysis of the SGA severity and achievement, as well as assessing a potential interaction between SGA birthweight and preterm birth.

## Methods

### Study design

Information was available through the Statistics New Zealand Integrated Data Infrastructure (IDI), a collection of datasets sourced from various governmental and non-governmental organisations that can be linked at the individual level based on unique identifiers. This is a de-identified database, and all information published has undergone rigorous checking to ensure anonymity [14].

### Study population

The birth population of interest was from Jan 1, 1998, to Dec 31, 2005. This ensures that children in the cohort have had the opportunity to complete secondary school, with conservative estimates suggesting most children graduate at the age of 18 (e.g. in 2023 for the youngest in the cohort). All singleton live births were considered eligible; multiple births were excluded (*n* = 14,443). Children missing gestational age, birthweight or infant sex were excluded as it prevented the calculation of birthweight percentiles (*n* = 6784). Despite compulsory schooling, some participants will spend significant time away from the classroom, generally due to unjustified absences (truancy).

If a participant was out of the country during the school year, they would normally be sitting secondary school assessments (ages 16 to 18); they were excluded from the study population as they were considered to no longer be resident in New Zealand (*n* = 56,688). Children who died prior to their 16th birthday (*n* = 3657) or were alive but could not be linked to education records (*n* = 6864) were also excluded. Children missing from education records may be home-schooled, in special-education or have received an exemption to leave school. We do not have the information required to estimate the number of missing data points for each reason. The rate of missingness is low, and selection bias is unlikely to impact the study findings. It is likely that the inclusion of these individuals would slightly reduce the overall level of achievement; however, we expect this will have little association with the estimated effect of SGA. The final population was 372,519 (SGA [25,341, (6.8%)]; AGA [347,178]). Children with congenital abnormalities were not identified and thus remained in the study population. These only constitute approximately 4% of the sample population (based on prevalence data from the Plunket National Child Health Study from 1990 to 1991) [15].

### Data linkage

The IDI is constructed around a central ‘spine’ of individuals who have ever been resident in Aotearoa, New Zealand. The aim is to maximise the coverage of the New Zealand population using minimal data sources: births, tax registrations and migration data. Following the construction of the spine, all other data sources in the IDI are linked to the central ‘spine’. Individual records were probabilistically linked by name, date of birth, and sex, and encrypted to ensure anonymity. Linkage does not guarantee it is a true match. Some records will be incorrectly linked; however, this is estimated to be the case in less than 1% of links [16]. Data from the IDI must be randomly rounded prior to being output to ensure anonymity. As a result, the counts presented in this paper may have minor differences.

### Exposures

Small for gestational age was defined as a birthweight in the lowest 10th percentile on the New Zealand sex-specific birthweight standard [1]. Secondary analysis categorised severe small for gestational age as a birthweight below the 3rd percentile, and moderate SGA was a birthweight between the 3rd and 10th percentile. Gestational age was categorised as very preterm (< 32 weeks’ gestation), preterm (32 to < 37 weeks’ gestation), and term birth (37 + weeks’ gestation) to explore interactions with small for gestational age. For sensitivity analyses, birthweight percentiles were recalculated on the New Zealand Customised Standard [17].

### Outcome

The outcome of interest was the highest qualification attained in secondary school (as per the New Zealand Qualification Framework) [18]. Secondary school achievement is measured using certificate attainment. These certificates are achieved by passing a series of assessments and exams in chosen subjects across the school year. There are three certificates: level one, level two, and level three, attempted sequentially across the last three years of secondary school (ages 16–18). Children in New Zealand are required by law to remain at school until the age of 16; thus, most students will attempt at least their level one certificate. Level two and three certificates are optional; however, a level two certificate is the minimum requirement for entry to tertiary education. University entrance is generally gained concurrently with the level three certificate, and early entry into university (following a level two qualification) requires exemption by individual tertiary providers. Obtaining a qualification is not a requirement for leaving secondary school.

### Statistical analysis

The chi-square test of independence was used to assess differences in the distribution of variables between the SGA and AGA populations. Due to a violation of the proportional odds assumption of ordinal logistic regression, multinomial logistic regression was used. To emulate this and allow for direct relative risk estimation, we used a series of log-binomial models. These derive the relative risk of achieving the highest level of no qualification, a level one qualification, or a level two qualification compared to a level three qualification in secondary school. High-dimensional models had issues with convergence when using the default Newton–Raphson optimisation. Coefficients for these models were estimated using the quasi-Newton algorithm. Confidence intervals were derived using the Huber-White sandwich variance estimator. The potential interaction between SGA birthweight and gestational age categories was performed by including a product term (SGA (Yes/No) × Gestational age (3 categories)) in a multivariable logistic regression model (with 2 degrees of freedom) predicting educational achievement. Statistical significance of the interaction term was assessed using a likelihood ratio test comparing models with and without the product term. While multiple children from the same family may have been enrolled in the cohort, individual-level data were analysed independently without adjusting for sibling-level clustering.

Each model was adjusted for a variety of a priori confounding factors: ethnicity (prioritised based on Ministry of Health framework [19], categorical), deprivation (New Zealand Deprivation Index of 2001 [20], decile categories which ascend from low deprivation to high deprivation), parity (categorical), maternal and paternal age at delivery (5-year increments, categorical), and maternal and paternal qualification (ordinal based on NQF levels of qualification where no qualification is considered the lowest, and postdoctoral degree is considered the highest). This study has attempted to adjust for all known confounders routinely available through the data register. However, there remains the potential of residual confounding through maternal health factors (e.g. chronic stress or anxiety) or educational factors (e.g. parents' perspectives on the importance of education).

Statistical analyses were performed using SAS version 9.4 software (SAS Institute Inc.). A *p*-value < 0.05 was assessed as defining statistical significance.

### Missing data

Information on SGA birthweight and educational achievement was available for all individuals in the dataset. Missing covariate information has been provided in Table 1. Analysis included missing categories for variables where required. Estimated effects for the missing category were not included in the odds ratio table. The number of observations missing for each variable can be found in Table 1.

### Sensitivity analysis

Small for gestational age was redefined using an updated birthweight standard, the New Zealand Customised Birthweight Standard [17]. This aims to improve the discrimination between constitutionally small infants and those with fetal growth restriction. A subgroup analysis of all study participants born in 2003 to 2005 was reanalysed using the same analyses described above. Analysis was restricted to a subset of the original population as the necessary information for customised birthweight centile calculation was not available for the entire study period (i.e., maternal height and weight were not routinely recorded until 2003). The adjusted subgroup analysis did not control for the effects of ethnicity or parity, as these factors were already accounted for in the centile calculation.

*Ethical approval.*

This project involved data sourced from the Statistics New Zealand Integrated Data Infrastructure (IDI). Access to IDI data for ‘public good’ research is available to researchers by application. The application to access data for this research was approved on 12/04/2024 by the University of Auckland Human Participants Ethics Committee (UAHPEC27332). The UAHPEC standards were designed to meet high international ethical benchmarks, which align with the principles of the Declaration of Helsinki. Consent to Participate declaration is not applicable in accordance with the legislation governing the Integrated Data Infrastructure.

## Results

**Fig. 1:**
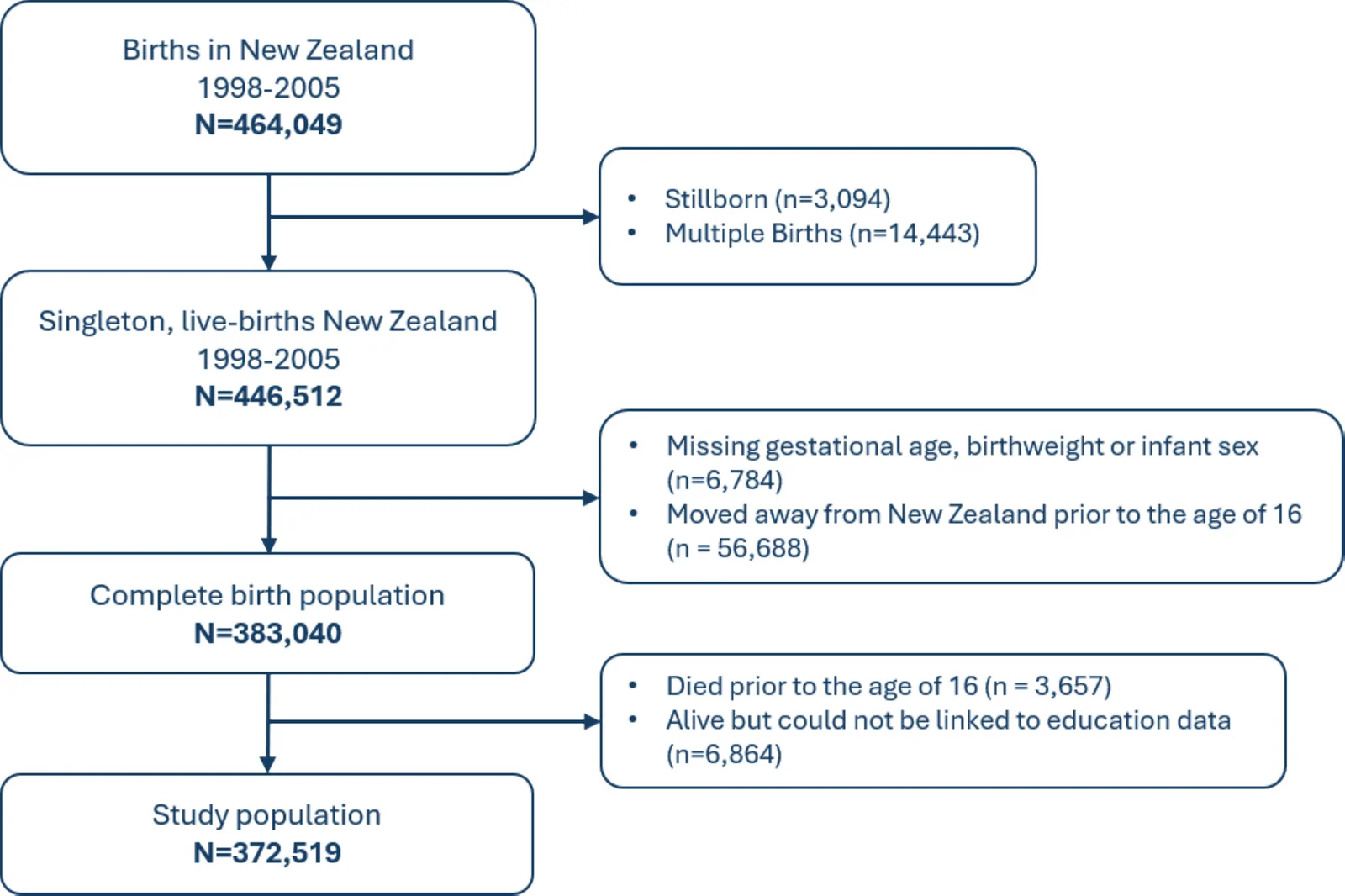
Flow diagram of study population

From Jan 1, 1998, to Dec 31, 2005, 464,049 children were born in New Zealand. A total of 446,512 singleton live births were eligible for inclusion. Of these, 6784 were missing any combination of gestational age, birthweight or sex information. A further 67,200 had either moved overseas, died or were unable to be linked to education records. Thus, the total number of children included in the final study population was 372,519 (Fig. 1). Of those identified, 6.8% (*n* = 25,341) were found to be SGA at birth.

The comparison of demographic characteristics of SGA and AGA infants is included in Table 1. The demographic characteristics differed significantly between the SGA and AGA populations. Of particular note is the greater number of children born SGA with teenage mothers at the time of birth (7.3% vs 5.1%, *p* < 0.001), teenage fathers at the time of birth (3.1% vs 2.1%, *p* < 0.001), nulliparity (65.7% vs 50.2%, *p* < 0.001), and Māori ethnicity (38.8% vs 30.4%, *p* < 0.001). The average age of participants at the time of data extraction was 22 years 6 months.

**Table 1:** Demographic characteristics of the study population

	AGA (*n *= 347,178)^a^	SGA (*n *= 25,341)^a^	*p*-value
	*N (col %)*	*N (col %)*	
**Infant sex**			*p* = 0.0771
Female	169,434 (48.8)	12,222 (48.2)	
Male	177,744 (51.2)	13,119 (51.7)	
**Ethnicity**			*p* < 0.0001
Asian	22,167 (6.3)	3045 (12.0)	
European	179,925 (51.8)	10,452 (41.2)	
MELAA	2073 (0.6)	144 (0.5)	
Maori	105,483 (30.3)	9834 (38.8)	
Other	4788 (1.3)	309 (1.2)	
Pasifika	32,742 (9.4)	1551 (6.1)	
**Gestational age**			*p* < 0.0001
Very Preterm Birth	2775 (0.8)	234 (0.9)	
Preterm Birth	16,908 (4.8)	1389 (5.4)	
Term Birth	327,495 (94.3)	23,718 (93.6)	
**Deprivation**			*p* < 0.0001
Decile 1	25,428 (7.3)	1344 (5.3)	
Decile 2	25,518 (7.3)	1287 (5.0)	
Decile 3	27,018 (7.7)	1590 (6.2)	
Decile 4	27,108 (7.8)	1659 (6.5)	
Decile 5	28,287 (8.1)	1872 (7.3)	
Decile 6	29,229 (8.4)	2037 (8.0)	
Decile 7	30,921 (8.9)	2346 (9.2)	
Decile 8	33,195 (9.5)	261 (10.5)	
Decile 9	36,759 (10.5)	3408 (13.4)	
Decile 10	42,966 (12.3)	4299 (16.9)	
Missing	40,752 (11.7)	2838 (11.2)	
**Parity**			*p* < 0.0001
0	174,438 (50.2)	16,668 (65.7)	
1	103,905 (29.9)	5208 (20.5)	
2	42,924 (12.3)	1923 (7.5)	
3 +	25,914 (7.4)	1545 (6.1)	
**Maternal age at delivery**			*p* < 0.0001
< 20	17,628 (5.0)	1860 (7.3)	
20–24	53,325 (15.3)	4857 (19.1)	
25–29	83,268 (23.9)	6066 (23.9)	
30–34	105,447 (30.3)	6561 (25.8)	
35–39	57,564 (16.5)	3549 (14.0)	
> = 40	12,603 (3.6)	945 (3.7)	
Missing	17,340 (4.9)	1500 (5.9)	
**Paternal age at delivery**			*p* < 0.0001
< 20	7314 (2.1)	783 (3.0)	
20–24	32,910 (9.4)	2862 (11.2)	
25–29	61,860 (17.8)	4593 (18.1)	
30–34	95,661 (27.5)	6093 (24.0)	
35–39	73,431 (21.1)	4515 (17.8)	
> = 40	41,772 (12.0)	2859 (11.2)	
Missing	34,239 (9.8)	3636 (14.3)	
**Maternal qualification at birth**			*p* < 0.0001
No Qualification	57,729 (16.6)	6054 (23.8)	
HS Certificate	118,347 (34.0)	7809 (30.8)	
Certificate/Diploma	52,968 (15.2)	3174 (12.5)	
Bachelor’s degree	49,254 (14.1)	2619 (10.3)	
Postgraduate degree +	9765 (2.8)	207 (5.2)	
Missing	59,121 (17.0)	5157 (20.3)	
**Paternal qualification at birth**			*p* < 0.0001
No Qualification	61,086 (17.6)	5532 (21.8)	
HS Certificate	82,317 (23.7)	5193 (20.4)	
Certificate/Diploma	69,087 (19.9)	3912 (15.4)	
Bachelor’s degree	33,495 (9.6)	1719 (6.7)	
Postgraduate degree +	11,985 (3.4)	648 (2.5)	
Missing	89,211 (25.7)	8340 (32.9)	

^a^Counts have been randomly rounded to adhere to the IDI output requirements set by Statistics New Zealand

Over the study period, 20,943 (5.6%) children did not attain a formal secondary school qualification, 54,204 (14.4%) attained at most a level one qualification, 116,001 (30.9%) attained at most a level two qualification, and 181,374 (49.1%) finished secondary school with the maximum level three qualification. When comparing qualification attainment by SGA status using a log-binomial model, the level of educational achievement differed slightly between SGA and AGA children (*p* < 0.001). After adjusting for confounding, the relative risk of achieving no formal qualification compared to a level three qualification was 1.36 times greater for the SGA group compared to their AGA peers (95% CI, 1.25–1.47). This risk was smaller but remained statistically significant with increasing achievement level. Children born SGA were at a 1.15 (95% CI 1.10–1.20) times greater risk of only achieving at most a level one qualification and a 1.07 (95% CI 1.04–1.10) times greater risk of only achieving a level two qualification (Table 2). The estimated adjusted relative risk of confounding variables is presented in Supplementary Table 1.

**Table 2:** Counts and relative risk of certificate level attainment in secondary school, by population small for gestational age birthweight

	SGA	AGA	Relative risk (95% CI)		Crude absolute risk difference (%)
	*N* (col %)	*N* (col %)	Unadjusted	Adjusted^b^	
No qualification	2082 (8.2)	18,861 (5.4)	1.64 (1.58, 1.71)	1.36 (1.25, 1.47)	2.8 (2.5, 3.2)
Level One	4578 (18.1)	49,626 (14.3)	1.34 (1.30, 1.37)	1.15 (1.10, 1.20)	3.8 (3.3, 4.3)
Level Two	8034 (31.7)	107,967 (31.1)	1.11 (1.09, 1.13)	1.07 (1.04, 1.10)	0.60 (0.0, 1.2)
Level Three	10,647 (42.0)	170,727 (49.2)	1.00 (Reference)	1.00 (Reference)	− 7.2 (− 7.8, − 6.6)

^b^ Adjusted for ethnicity, deprivation, parity, maternal and paternal age at delivery, and maternal and paternal qualification

Severity of SGA birthweight shows larger effect sizes for severe SGA (< 3%) than moderate SGA (3–10%). After adjustment for confounding, children born severely SGA were at a 1.48 times greater risk of attaining no formal qualification (95% CI 1.29–1.70), a 1.26 times greater risk of attaining at most a level one qualification (95% CI 1.16–1.36) and a 1.07 times greater risk of attaining a level two qualification compared to their AGA peers (95% CI 0.02–1.13). The respective risks for moderate SGA were 1.30 (95% CI, 1.18–1.44), 1.11 (95% CI, 1.04–1.17), and 1.07 times (95% CI, 1.03–1.10) greater than those for AGA children (Table 3).

**Table 3:** Counts and relative risk of certificate level attainment in secondary school, by SGA severity at birth

	Participant	Relative risk (95% CI)		Crude absolute risk difference (%)
	*N* (col %)	Unadjusted	Adjusted^c^	
**Severe SGA**				
No Qualification	651 (8.9)	1.82 (1.69, 1.95)	1.48 (1.29, 1.70)	3.5 (2.8, 4.2)
Level One	1446 (19.7)	1.46 (1.40, 1.52)	1.26 (1.16, 1.36)	5.4 (4.5, 6.3)
Level Two	2301 (31.3)	1.13 (1.10, 1.17)	1.07 (1.02, 1.13)	0.2 (− 0.9, 1.3)
Level Three	2955 (40.2)	1.00 (Reference)	1.00 (Reference)	− 9.0 (− 10.4, − 7.9)
**Moderate SGA**				
No Qualification	1431 (8.0)	1.57 (1.50, 1.65)	1.30 (1.18, 1.44)	2.6 (2.2, 3.0)
Level One	3129 (17.4)	1.28 (1.25, 1.32)	1.11 (1.04, 1.17)	3.1 (2.5, 3.7)
Level Two	5736 (31.9)	1.10 (1.08, 1.12)	1.07 (1.03, 1.10)	0.8 (0.1, 1.5)
Level Three	7692 (42.8)	1.00 (Reference)	1.00 (Reference)	− 6.4 (− 7.1, − 5.6)

^c^Adjusted for ethnicity, deprivation, parity, maternal and paternal age at delivery, and maternal and paternal qualification

**Fig. 2:**
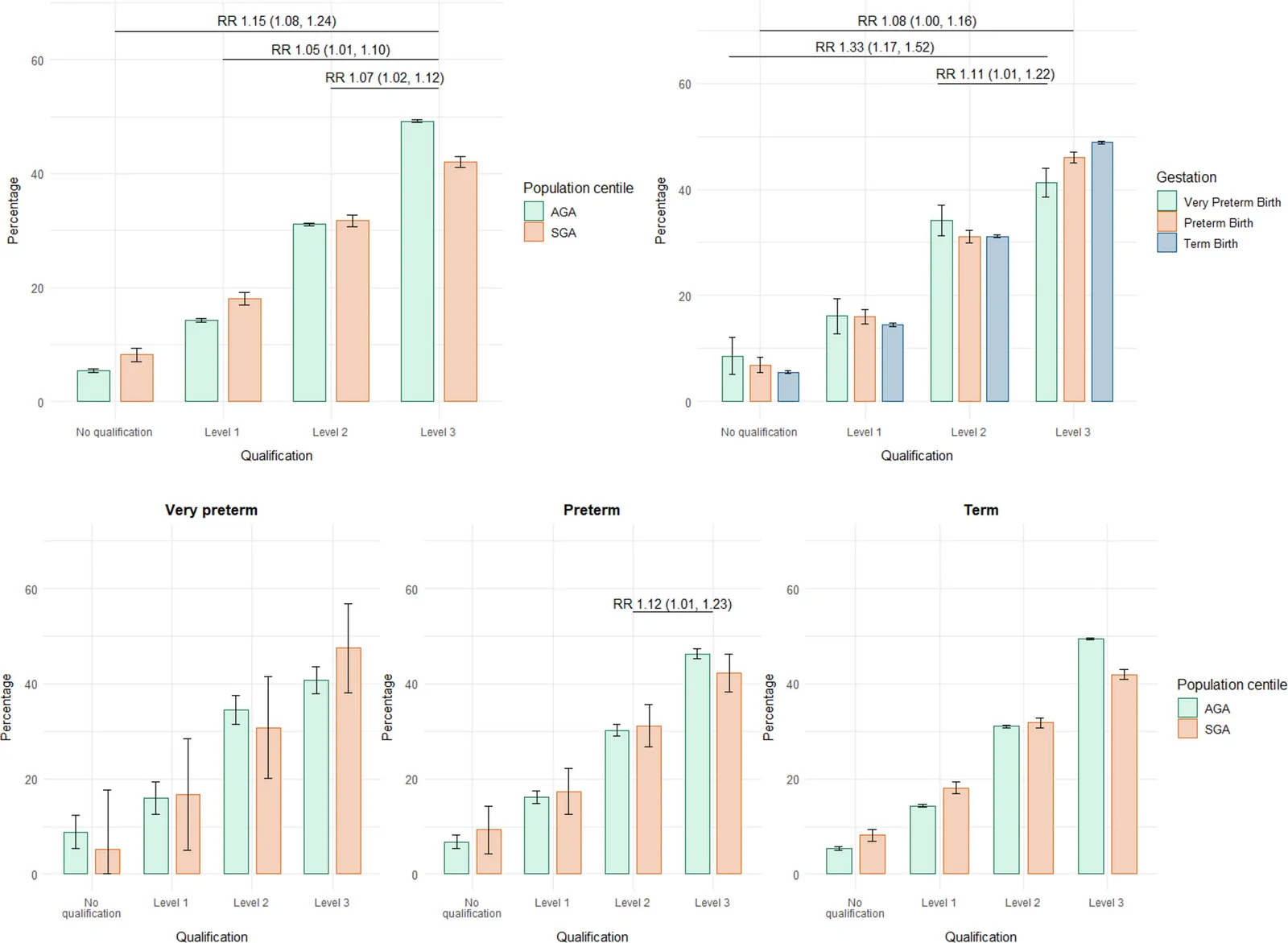
Distribution of qualification attainment by population birthweight percentiles and gestational age. Confidence intervals for estimates are presented as error bars

There is a positive multiplicative interaction between SGA birthweight and gestational age categories (*p* < 0.0001). When stratifying by gestational age categories, children born SGA born at term were at an elevated risk of attaining a no qualification, a level one qualification or a level two qualification, compared to AGA term children (aRR: 1.43, 95% CI: 1.26, 1.63 [no qualification]; aRR: 1.12, 95% CI: 1.04, 1.21 [level one]; aRR: 1.05, 95% CI: 1.00, 1.10 [level two]). There was no statistically significant effect of SGA birthweight in the preterm or very preterm group (see Table 4 for relative risk and absolute risk difference estimates). The independent effect of being born very preterm on attaining each qualification is comparable to the effect of SGA birthweight (aRR: 1.33, 95% CI: 1.17, 1.52 [no qualification]; aRR: 1.05, 95% CI: 0.94, 1.17 [level one]; aRR: 1.11, 95% CI: 1.01–1.22 [level two]). Preterm birth has a marginally smaller independent association with educational achievement than SGA birthweight, with only the effect of preterm birth on no formal qualification reaching statistical significance (aRR: 1.08, 95% CI: 1.00, 1.16 [no qualification]) (Fig. 2). The joint exposure resulted in a relative risk that exceeded the expected product of their individual effects—meaning the two factors amplify each other’s impact rather than acting independently, confirming a synergistic interaction.

**Table 4:** Relative risk of certificate level attainment in secondary school, by SGA status and gestational age at birth

	SGA	AGA	Relative risk (95% CI)		Crude absolute risk difference (%)
	*N* (col %)	*N* (col %)	Unadjusted	Adjusted^d^	
**Very preterm birth**					
No qualification	12 (5.1)	246 (8.9)	0.59 (0.35, 0.99)	0.80 (0.36, 1.77)	− 3.8 (− 6.7, − 0.8)
Level One	39 (16.7)	444 (16.0)	0.94 (0.71, 1.24)	0.84 (0.45, 1.57)	0.70 (− 4.3, 5.7)
Level Two	72 (30.8)	954 (34.4)	0.86 (0.71, 1.03)	0.90 (0.64, 1.27)	− 3.6 (− 9.8, 2.6)
Level Three	111 (47.4)	1128 (40.7)	1.00 (Reference)	1.00 (Reference)	6.7 (0.1, 13.3)
**Preterm birth**					
No qualification	129 (9.3)	1131 (6.8)	1.43 (1.21, 1.69)	1.34 (0.93, 1.93)	2.5 (0.9, 4.1)
Level One	240 (17.3)	2688 (16.1)	1.14 (1.02, 1.28)	1.07 (0.87, 1.32)	1.2 (− 0.9, 3.3)
Level Two	432 (31.2)	5049 (30.2)	1.06 (0.98, 1.14)	1.02 (0.89, 1.16)	1.0 (− 1.5, 3.5)
Level Three	585 (42.2)	7839 (46.3)	1.00 (Reference)	1.00 (Reference)	− 4.7 (− 7.4, − 2.0)
**Term birth**					
No qualification	1938 (8.2)	17,487 (5.3)	1.67 (1.60, 1.75)	1.43 (1.26, 1.63)	2.9 (2.5, 3.3)
Level One	4296 (18.1)	46,491 (14.3)	1.35 (1.32, 1.39)	1.12 (1.04, 1.21)	3.9 (3.4, 4.4)
Level Two	7530 (31.8)	101,736 (31.1)	1.12 (1.10, 1.14)	1.05 (1.00, 1.10)	0.7 (0.1, 1.3)
Level Three	9948 (42.0)	161,757 (49.4)	1.00 (Reference)	1.00 (Reference)	− 7.4 (− 8.1, − 6.7)

^d^Adjusted for ethnicity, deprivation, parity, maternal and paternal age at delivery, and maternal and paternal qualification

Importantly, the results attained in the primary analysis were closely replicated in the sensitivity analysis of customised SGA and educational attainment (Supplementary Table 2) (aRR: 1.31, 95% CI: 1.20, 1.42 [no qualification]; aRR: 1.14 95% CI: 0.08, 1.20 [level one], aRR: 1.05, 95% CI: 1.01, 1.08 [level two]).

## Discussion

### Principal findings

We aimed to assess the risk of low educational attainment in secondary school for SGA children, using nationally representative administrative health and education data in New Zealand. We found that SGA birthweight was significantly associated with secondary school achievement. After adjustment for confounding, SGA were at the greatest risk of achieving no formal qualification compared to a level three qualification. The risk of attaining a level one or two qualification compared to a level three qualification was statistically significant, but smaller in effect size. The estimated effects in this paper, while statistically significant, were relatively small in magnitude. Despite this, with approximately 10% of the population born SGA, the finding is important at the population level.

The present study also demonstrates that the severity of SGA birthweight modifies the relationship with education. Children with severe SGA birthweight (< 3%) were at the greatest risk of low attainment. This is consistent with previous studies that find decreasing birthweight percentile is associated with significantly increased risk of low school achievement [11, 13]. It is suspected that this is the result of an increase in sensitivity for detecting FGR using a 3rd percentile threshold for SGA, and decreasing the threshold for SGA increases Type II error related to FGR detection. Research has suggested that fetal growth restriction, rather than constitutional smallness, drives low brain development and subsequent educational underachievement [13].

Stratified analyses of this study cohort revealed that the effects of SGA birthweight were limited to those born at term. There was no difference in the risk of low achievement for children born very preterm or preterm SGA compared to their AGA peers born at a similar gestational age. Furthermore, the independent effect of very preterm birth and preterm birth on educational outcomes is comparable in effect size to that of SGA birthweight. Therefore, while studies have emphasised the relative importance of gestational age, this paper has demonstrated that SGA birthweight at term is equally important, particularly as SGA has a higher prevalence in the population.

The biological mechanisms for this have not been well established. Studies have suggested that antenatal steroid administration in very preterm infants can improve brain development [21–23]; however, the effect of antenatal steroid administration on long-term neurodevelopmental outcomes has not been demonstrated [22, 23]. Another possible explanation is the increased prevalence of FGR among very preterm and preterm-AGA infants. This may lead to postnatal growth patterns and neurodevelopmental risks resembling those of infants born SGA. The associations reported were attenuated dramatically with adjustment for confounders. This suggests that social determinants were the predominant cause for both SGA birthweight and low educational achievement. For example, the risk of low attainment increases with deprivation at the time of birth, young parental age at delivery and parents with no formal qualifications. The estimated effect sizes for these variables were greater than the independent effect of being small for gestational age, indicating that environmental factors drive underachievement to a comparable or greater extent than SGA birthweight.

### Interpretation

Many studies have explored the association between SGA birthweight and education; few have used school outcomes as a measure of academic performance. Those that do differ greatly in the specific outcome of interest (e.g. school entry, school grades, baccalaureate attainment) [6, 7, 11]. Several studies find that children born SGA have a greater risk of lower school achievement [6, 11]. Those that fail to detect differences are often underpowered due to small sample sizes [7]. Of particular interest is a study by Gustafsson (2023). This study used a national birth register and compulsory schooling data for over two million children. Gustafsson et al. found that term-born children born SGA were 1.41 times as likely to have a final average grade below the 10th percentile and 1.15 times as likely to have grades between the 10th and 25th percentile compared to AGA children (after adjustment for confounders) [5]. These estimates are like those obtained in this study. However, the present study was also able to demonstrate a dose–response relationship between SGA severity and risk of low achievement, as well as an interaction with preterm birth.

The current paper also explored the effect of SGA severity on educational achievement. Decreasing the threshold for defining SGA to the 3rd percentile aims to capture those with the greatest degree of growth restriction and increases the likelihood that low birthweight has a physiological cause. As mentioned previously, this paper found that children born severely SGA were at an elevated risk of low attainment compared to those with moderate-SGA birthweight. This has only been demonstrated in one previous study, which found an inverse relationship between birthweight percentile and risk of low achievement [11]. Other studies have aimed to capture the effect of FGR using direct diagnoses such as the ponderal index or serial ultrasound measurements [6, 13, 24]. However, only one looked at the effect of FGR on school achievement (as opposed to intelligence); this study found the risk of FGR children not achieving their baccalaureate was comparable to the severe-SGA effect of certificate attainment shown in the present study [6].

There were many suspected mechanisms for the association between SGA birthweight and educational achievement. The Barker Hypothesis suggests that an adverse intrauterine environment during critical periods of embryonic and fetal development can permanently program the structure and physiology of the developing fetus [25]. While brain-sparing (a prioritisation of blood flow to the brain) has been shown to protect the brain in times of reduced nutrients, early nutritional insults can limit brain development [26]. Alternatively, the Developmental Origins of Health and Disease paradigm extends this also to consider environmental factors such as maternal stress as a possible cause for poor outcomes. Both could result in epigenetic changes that could manifest as lower cognitive function [27].

### Study strengths and limitations

The estimates obtained in this paper were representative of the New Zealand population, particularly for births occurring in the late 1990 s and early 2000s. Since then, there has been a rapid advancement in prenatal and neonatal care that could alter the observed effect. This change is difficult to estimate, as multiple factors will occur simultaneously. First, improvements in preterm care have decreased the gestational age of viable neonates, inadvertently increasing the size of the fetal growth restriction population and those at a greater risk of impaired educational achievement.

There is the issue of being unable to diagnose individuals with FGR. Fetal growth restriction diagnosis has been demonstrated to be challenging. The most reliable method appears to be a combination of Doppler studies and serial ultrasound measurements [28]. However, this is only offered to mothers in New Zealand who present to antenatal appointments with one major or three minor risk factors for FGR [28]. This information is not routinely collected and is not available through the IDI. Instead, this paper used SGA birthweight as a measure of growth compromise during fetal development. However, this is an imperfect measure and will not completely capture the FGR population. As FGR children were more compromised during fetal development than their constitutionally small peers, we expect the true effect of FGR on achievement to be greater than the observed effect outlined in this paper.

Where most countries quantify secondary school achievement as an individual’s performance on a final bursary test, New Zealand has a cumulative approach to secondary school achievement (qualifications were accumulated across several years) [29]. As a result, this paper was able to compare SGA and AGA achievement on certificates attained across three consecutive years. Interestingly, there is evidence that SGA is not only a risk factor for not achieving a formal qualification, but the risk persists with increasing certificate level. This effect is present even though New Zealand school qualifications (National Certificate of Educational Attainment) were personalised to focus on the student’s strengths and future aspirations, encouraging achievement.

The New Zealand qualification system has remained consistent from 2011 to the present day, during which time those in this cohort would have been gaining their school qualifications. There have been minor changes in subject availability; however, most assessments have retained the same structure. New Zealand is a developed country with government-funded health care and education systems. Schooling is compulsory to 16 years of age. Thus, we believe the results from this paper are applicable to similar Western societies beyond New Zealand. Caution should be taken when applying findings to developing countries where macro-undernutrition has a more substantial influence on SGA birthweight, and in countries where education is not widely available. Some variation in findings may be present in countries with different educational assessment methods.

Many current studies suffer from methodological issues such as a limited sample size and the inability to control for a wide range of confounding factors. This study is sufficiently powered to adjust for numerous confounding effects, explore the attenuation of estimates when comparing small for gestational age severity and the potential interaction between small for gestational age birthweight and preterm birth. This paper also benefited from the wide range of individualised information that can be used for the adjusted analysis, which is not often collected in cohort or case–control studies. A limitation of this study is the inability to cluster observations at the family level. Therefore, this analysis did not account for shared genetic or environmental variance among siblings. Consequently, standard errors may be slightly underestimated if strong familial clustering was present. This paper also does not provide information on the demographics associated with those included vs excluded from the study population. However, most individuals excluded had moved overseas prior to the age of 16 and therefore were not part of the target study population. Those excluded due to missing information required for birthweight percentile calculation or missing linkage with education records constitute a small proportion of the study population; exclusion is unlikely to influence estimated risks.

Recall bias is mitigated when using the IDI. Most variables used in this study were recorded by the participant at a certain time (census) or by a governmental organisation at the time of the event (incarceration, death or benefit support). This information is not immune to recording error. Erroneous observations were removed where identifiable, as the sample size was sufficient to risk losing a small number of observations suspected to be incorrect. Long-term outcomes can be assessed in the IDI without fear that those lost to follow-up represent a critical population or that the sample size decrease will impair study power. This makes it possible to explore outcomes that occur well into adulthood. Linkage errors within the IDI need to be considered. Errors can be caused by incorrect linkage between two individuals or missing linkage for the same individual. This can lead to information bias and selection bias, particularly since linkage errors are not evenly distributed across groups of individuals. The Ministry of Education (MOE) dataset has a 1.8% false positive rate. Previous research has indicated that linkage error in MOE data does not appear to have much impact on results [16].

## Conclusion

Our results, alongside previous publications, suggest that those born SGA are significantly less likely to achieve high school qualifications compared to their AGA peers born preterm or term. This risk increases with the severity of SGA. Adjustment for confounders decreases the effect size of SGA birthweight; however, it remains significant in the analysis. This suggests that SGA birthweight may have an independent risk factor for educational attainment; however, social and environmental factors also influence academic achievement. This is an observational study, which prevents conclusions of causality. Given the high prevalence of SGA in the population, improvements in educational standards for these children could have societal effects.

## Supplementary Information

Below is the link to the electronic supplementary material.

ESM 1 (DOCX 24.9 KB)

## Data Availability

The data that supports the findings of this study are available in the Statistics New Zealand Integrated Data Infrastructure, but restrictions apply to the availability of these data. The data is available to those with IDI accreditation.
