# Supplementary material for: A large national cohort study exploring the association between small for gestational age birthweight and educational achievement in secondary school
Source: Eur J Pediatr. 2026 Sep 2;185(9):712. doi: 10.1007/s00431-026-07372-w (PMC13538170; doi:10.1007/s00431-026-07372-w)
Supplement: Supplementary file 1 [file 431_2026_7372_MOESM1_ESM.docx]

| **Supplementary Table 1** Adjusted relative risks of secondary school certificate attainment, by population SGA birthweight | | | |
| --- | --- | --- | --- |
|  | **No Qualification** | **Level One** | **Level Two** |
|  | *aRR (95% CI)* | *aRR (95% CI)* | *aRR (95% CI)* |
| **Ethnicity** |  |  |  |
| Maori | 1.66 (1.57, 1.77) | 0.51 (0.38, 0.67) | 0.85 (0.76, 0.96) |
| Pasifika | 0.77 (0.69, 0.86) | 0.60 (0.57, 0.64) | 0.81 (0.79, 0.84) |
| Asian | 1.23 (1.12, 1.36) | 0.38 (0.35, 0.42) | 0.54 (0.51, 0.56) |
| MELAA | 1.00 (0.69, 1.45) | 0.78 (0.76, 0.80) | 0.90 (0.89, 0.92) |
| European | 1.00 (Reference) | 1.00 (Reference) | 1.00 (Reference) |
| Other | 1.21 (0.96, 1.51) | 0.92 (0.82, 1.03) | 1.01 (0.95, 1.07) |
| **Deprivation** |  |  |  |
| Decile 1 | 1.00 (Reference) | 1.00 (Reference) | 1.00 (Reference) |
| Decile 2 | 1.07 (0.96, 1.20) | 0.99 (0.94, 1.05) | 1.01 (0.98, 1.05) |
| Decile 3 | 0.92 (0.82, 1.03) | 0.96 (0.90, 1.02) | 0.97 (0.94, 1.00) |
| Decile 4 | 0.96 (0.86, 1.08) | 0.96 (0.91, 1.02) | 0.98 (0.95, 1.01) |
| Decile 5 | 0.98 (0.88, 1.10) | 0.95 (0.90, 1.01) | 1.01 (0.97, 1.04) |
| Decile 6 | 1.01 (0.90, 1.13) | 0.94 (0.89, 1.00) | 0.96 (0.93, 1.00) |
| Decile 7 | 1.03 (0.92, 1.15) | 0.95 (0.90, 1.01) | 0.97 (0.94, 1.01) |
| Decile 8 | 1.01 (0.90, 1.12) | 0.94 (0.89, 1.00) | 0.96 (0.93, 1.00) |
| Decile 9 | 0.96 (0.86, 1.07) | 0.94 (0.88, 0.99) | 0.98 (0.95, 1.01) |
| Decile 10 | 1.03 (0.92, 1.14) | 0.97 (0.91, 1.02) | 0.99 (0.96, 1.03) |
| **Parity** |  |  |  |
| 0 | 1.00 (Reference) | 1.00 (Reference) | 1.00 (Reference) |
| 1 | 1.05 (0.99, 1.12) | 1.08 (1.04, 1.11) | 1.04 (1.02, 1.06) |
| 2 | 1.19 (1.10, 1.29) | 1.21 (1.16, 1.26) | 1.09 (1.06, 1.12) |
| 3+ | 1.83 (1.67, 1.99) | 1.51 (1.44, 1.59) | 1.19 (1.15, 1.23) |
| **Maternal age at delivery** |  |  |  |
| <20 | 1.29 (1.10, 1.51) | 1.81 (1.69, 1.94) | 1.30 (1.24, 1.36) |
| 20-24 | 1.66 (1.50, 1.83) | 1.61 (1.53, 1.69) | 1.23 (1.20, 1.27) |
| 25-29 | 1.12 (1.04, 1.21) | 1.24 (1.19, 1.29) | 1.11 (1.09, 1.13) |
| 30-34 | 1.04 (0.96, 1.13) | 0.94 (0.90, 0.99) | 0.95 (0.93, 0.98) |
| 35-39 | 1.00 (Reference) | 1.00 (Reference) | 1.00 (Reference) |
| >=40 | 1.10 (1.00, 1.20) | 1.04 (0.96, 1.14) | 0.93 (0.88, 0.98) |
| **Paternal Age at delivery** |  |  |  |
| <20 | 1.29 (1.10, 1.51) | 1.17 (1.08, 1.26) | 1.08 (1.02, 1.14) |
| 20-24 | 1.18 (1.06, 1.31) | 1.23 (1.17, 1.30) | 1.08 (1.04, 1.11) |
| 25-29 | 1.10 (1.01, 1.19) | 1.14 (1.10, 1.19) | 1.07 (1.04, 1.09) |
| 30-34 | 0.99 (0.91, 1.07) | 0.94 (0.90, 0.98) | 0.97 (0.95, 0.99) |
| 35-39 | 1.00 (Reference) | 1.00 (Reference) | 1.00 (Reference) |
| >=40 | 1.10 (1.00, 1.20) | 1.00 (0.94, 1.05) | 1.00 (0.97, 1.03) |
| **Maternal qualification at birth** |  |  |  |
| No Qualification | 2.26 (2.07, 2.46) | 2.29 (2.17, 2.42) | 1.37 (1.33, 1.40) |
| HS Certificate | 1.13 (1.05, 1.23) | 1.55 (1.47, 1.63) | 1.19 (1.16, 1.22) |
| Certificate/Diploma | 1.08 (0.99, 1.19) | 1.37 (1.29, 1.45) | 1.13 (1.10, 1.16) |
| Bachelor’s degree | 1.00 (Reference) | 1.00 (Reference) | 1.00 (Reference) |
| Postgraduate degree + | 1.2 (1.03, 1.4) | 0.99 (0.88, 1.12) | 0.93 (0.88, 0.99) |
| **Paternal qualification at birth** |  |  |  |
| No Qualification | 1.73 (1.58, 1.90) | 2.76 (2.58, 2.96) | 1.64 (1.58, 1.69) |
| HS Certificate | 0.97 (0.89, 1.06) | 1.84 (1.72, 1.97) | 1.39 (1.35, 1.43) |
| Certificate/Diploma | 0.80 (0.73, 0.89) | 1.69 (1.58, 1.81) | 1.36 (1.32, 1.41) |
| Bachelor’s degree | 1.00 (Reference) | 1.00 (Reference) | 1.00 (Reference) |
| Postgraduate degree + | 1.18 (1.03, 1.35) | 1.15 (1.02, 1.29) | 0.97 (0.92, 1.03) |
| **Population SGA Status** |  |  |  |
| SGA | 1.36 (1.25, 1.47) | 1.15 (1.10, 1.20) | 1.07 (1.04, 1.10) |
| AGA | 1.00 (Reference) | 1.00 (Reference) | 1.00 (Reference) |

| **Supplementary Table 2** Counts and relative risk of certificate level attainment in secondary school, by customised small for gestational age birthweight | | | | |
| --- | --- | --- | --- | --- |
|  | SGA | AGA | Relative Risk (95% CI) | |
|  | N (col %) | N (col %) | Unadjusted | Adjusted^e^ |
| No qualification | 1776 (10.2) | 8250 (6.5) | 1.65 (1.58, 1.73) | 1.31 (1.20, 1.42) |
| Level One | 3477 (20.0) | 20199 (16.0) | 1.30 (1.27, 1.34) | 1.14 (1.08, 1.20) |
| Level Two | 6018 (34.7) | 45555 (36.1) | 1.07 (1.05, 1.09) | 1.05 (1.01, 1.08) |
| Level Three | 6093 (35.1) | 52188 (41.4) | 1.00 (Reference) | 1.00 (Reference) |

^e^ Adjusted for ethnicity, deprivation, parity, maternal and paternal age at delivery, and maternal and potential qualification
